# Functional Remodeling Associated With Language Recovery After Repetitive Transcranial Magnetic Stimulation in Chronic Aphasic Stroke

**DOI:** 10.3389/fneur.2022.809843

**Published:** 2022-03-07

**Authors:** Bing-Fong Lin, Shih-Ching Yeh, Yu-Chieh Jill Kao, Chia-Feng Lu, Po-Yi Tsai

**Affiliations:** ^1^Department of Biomedical Imaging and Radiological Sciences, National Yang Ming Chiao Tung University, Taipei, Taiwan; ^2^Department of Computer Science and Information Engineering, National Central University, Taoyuan, Taiwan; ^3^Institute of Biophotonics, National Yang Ming Chiao Tung University, Taipei, Taiwan; ^4^Department of Physical Medicine and Rehabilitation, Taipei Veterans General Hospital, Taipei, Taiwan; ^5^School of Medicine, National Yang Ming Chiao Tung University, Taipei, Taiwan

**Keywords:** aphasia, stroke, repetitive transcranial magnetic stimulation, fMRI, functional connectivity

## Abstract

**Background:**

Repetitive transcranial magnetic stimulation (rTMS) has shown promising efficacy in improving the language functions in poststroke aphasia. However, randomized controlled trials were lacking to investigate the rTMS-related neuroimaging changes underlying the therapeutic effects on language improvement in chronic aphasia.

**Objective:**

In this study, we aimed to evaluate the effects of low-frequency rTMS (LF-rTMS) on chronic poststroke aphasia. We hypothesized that the deactivation of the right pars triangularis could restore the balance of interhemispheric inhibition and, hence, facilitated the functional remodeling of language networks in both the hemispheres. Furthermore, the rTMS-induced functional reorganization should underpin the language recovery after rTMS.

**Methods:**

A total of 33 patients (22 males; age: 58.70 ± 13.77 years) with chronic stroke in the left hemisphere and nonfluent aphasia were recruited in this randomized double-blinded study. The ratio of randomization between the rTMS and sham groups is 17:16. All the patients received real 1-Hz rTMS or sham stimulation (placebo coil delivered < 5% of magnetic output with similar audible click-on discharge) at the right posterior pars triangularis for 10 consecutive weekdays (stroke onset to the first stimulation: 10.97 ± 10.35 months). Functional connectivity of language networks measured by resting-state fMRI was calculated and correlated to the scores of the Concise Chinese Aphasia Test by using the stepwise regression analysis.

**Results:**

After LF-rTMS intervention, significant improvement in language functions in terms of comprehension and expression abilities was observed compared with the sham group. The rTMS group showed a significant decrease of coupling strength between right pars triangularis and pars opercularis with a strengthened connection between right pars orbitalis and angular gyrus. Furthermore, the LF-rTMS significantly enhanced the coupling strength associated with left Wernicke area. Results of regression analysis showed that the identified functional remodeling involving both the hemispheres could support and predict the language recovery after LF-rTMS treatment.

**Conclusion:**

We reported the therapeutic effects of LF-rTMS and corresponding functional remodeling in chronic poststroke aphasia. Our results provided neuroimage evidence reflecting the rebalance of interhemispheric inhibition induced by LF-rTMS, which could facilitate future research in the refinement of rTMS protocol to optimize the neuromodulation efficacy and benefit the clinical management of patients with stroke.

## Introduction

Poststroke nonfluent aphasia is characterized by a disabling linguistic output resulting from the damage of language-related circuits in the brain. Patients with this condition have difficulty in performing basic communication tasks and recovering previous daily status (1). Studies have demonstrated the promising efficacy of repetitive transcranial magnetic stimulation (rTMS) as a non-invasive neuromodulatory intervention to treat stroke-related aphasia (2, 3). According to the rationale of paradoxical functional facilitation and the theory of interhemispheric imbalance in poststroke recovery, many randomized controlled studies have reported that the application of an inhibitory low-frequency rTMS (LF-rTMS) protocol over the contralesional homologous Broca area results in substantial language improvement (4–11). This finding provides an alternative option for patients with chronic aphasic who have less spontaneous recovery in the subacute phase.

Neuroplasticity associated with language recovery after aphasic stroke involves both the left and right hemispheres (12, 13). However, the roles of left and right hemispheres in language recovery have been under debate for decades (14). Previous studies revealed that the overactivation of the right pars triangularis impeded language performance after a stroke involving the left inferior frontal gyrus (5, 7, 14). A task functional MRI (fMRI) study of six patients with chronic poststroke aphasia found that the naming improvement after LF-rTMS treatment in chronic was correlated with remodeling of functional activation in bilateral networks (15). Increased activations in the left hemisphere, including the medial frontal, cingulate, supplementary motor, and fusiform gyrus areas, were observed during the naming task after the LF-rTMS treatment. However, the activation of the right pars triangularis (target of LF-rTMS) shifted to the right pars opercularis during the naming task (15). A meta-analysis study further concluded that the activation of perilesional and spared language areas in the left hemisphere facilitated the naming performance, whereas the activation of right language areas impeded the naming performance (16). However, the functional reorganization involves both the hemispheres after the inhibitory LF-rTMS remains inconclusive because of the lack of randomized controlled trials (RCTs). Therefore, a formal RCT with neuroimaging assessment is required to determine bilateral and intercortical changes after the rTMS treatment to elucidate the relationship between the network reorganization and language improvement in patients with aphasic stroke.

Resting-state fMRI (rs-fMRI) has been widely used to provide evidence of functional changes after stroke. Unlike task-based fMRI methods, rs-fMRI measures the intrinsic fluctuation of brain activities and provides a more flexible approach to investigate functional networks (17). Recent evidence indicates that aphasia is a network disorder with extensive involvement of altered functional connectivity (FC) rather than local damage caused by stroke (18). Accordingly, rs-fMRI analysis may help to comprehensively evaluate the disruption of language networks after stroke and remodeling with recovery. Several functional studies have reported that the secondary language circuits, including left spared cortices, perilesional tissues, and right homologous areas, might take over the language function after the primary language cortices are damaged (19, 20). The temporal shift of resting-state hemodynamic profiles may correspond to language performance after stroke (21). Moreover, lesion distribution may influence network remodeling and language performance after a stroke (19, 21). However, no RCT has evaluated the functional reorganization of language networks by using rs-fMRI after LF-rTMS treatment in patients with chronic poststroke aphasia.

In this RCT, we evaluated the therapeutic effects of LF-rTMS and associated functional remodeling in patients with chronic poststroke aphasia. Patients were randomly allocated to the rTMS or sham groups. We hypothesized that the deactivation of the right pars triangularis would restore the balance of interhemispheric inhibition, thus facilitating the functional remodeling of language networks in both the brain hemispheres. Furthermore, we anticipated that the rTMS-induced functional reorganization, which involves FC enhancement in the left hemisphere and corresponding modulation in the right hemisphere, could support language recovery after rTMS. Considering the predominant lateralization of language functions and aphasia as a network disorder, we investigated the modulation of the bilateral networks and effects on language improvement relevant to treatment-induced recovery.

## Materials and Methods

### Participants

**Figure 1:**
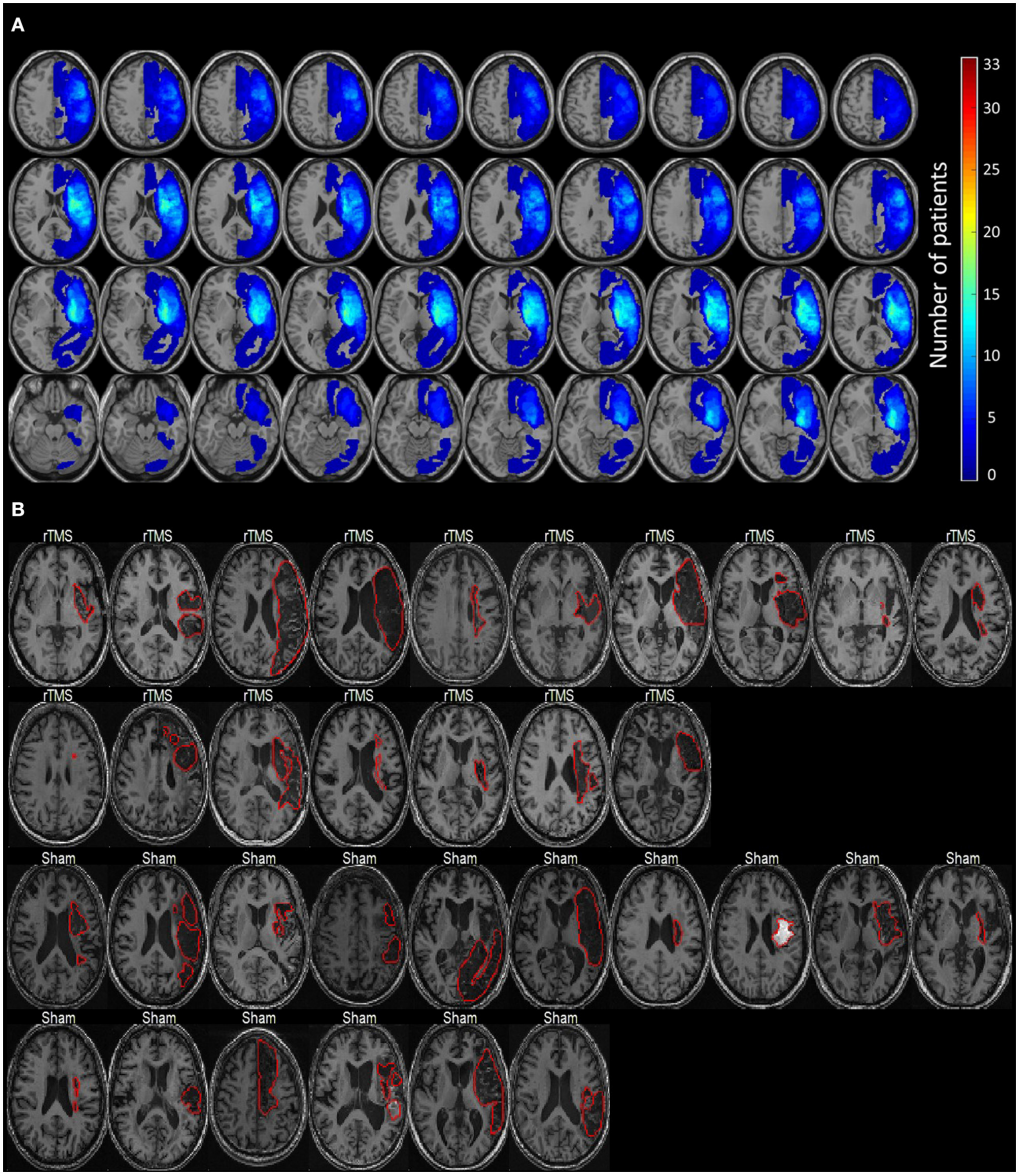
The lesion distribution of the study cohort. **(A)** The overlapping maps of lesion distribution among 33 patients. **(B)** The individual lesion pattern of each patient (delineated by the red contours). Lesions are all located in the left hemisphere.

A total of 54 patients who had chronic left hemispheric stroke with nonfluent aphasia and who were admitted to the stroke unit of a tertiary Medical Center were evaluated. The final cohort, including 33 patients with Chinese as their first language, was selected based on the following inclusion criteria: (1) a first ischemic stroke affecting the left territory of the middle cerebral artery as confirmed through MRI; (2) at least 3 months after stroke onset; (3) no history of dementia, affective disorders, or other neurodegenerative diseases; and (4) absence of rTMS contraindications. Nonfluent aphasia is characterized by anomia, short phrase length (0–5 words per breath unit), with or without auditory comprehension, or repetition impairment (22). Phrase length is defined as the average of the three longest meaningful utterances produced when a picture scene is described [e.g., “Cookie theft” in the Concise Chinese Aphasia Test (CCAT)] or when responding to an open-ended question. All the participants provided a written informed consent prior to study participation and the study protocol was in accordance with the 2008 Declaration of Helsinki and approved by the local institutional review board. This clinical trial was preregistered with the identifier of NCT03059225 (https://clinicaltrials.gov/ct2/show/NCT03059225). All the patients were right-handed and had scores of > 90 in the Edinburgh Inventory (23). Figure 1 presents the lesion distribution of the 33 recruited patients.

**Figure 2:**
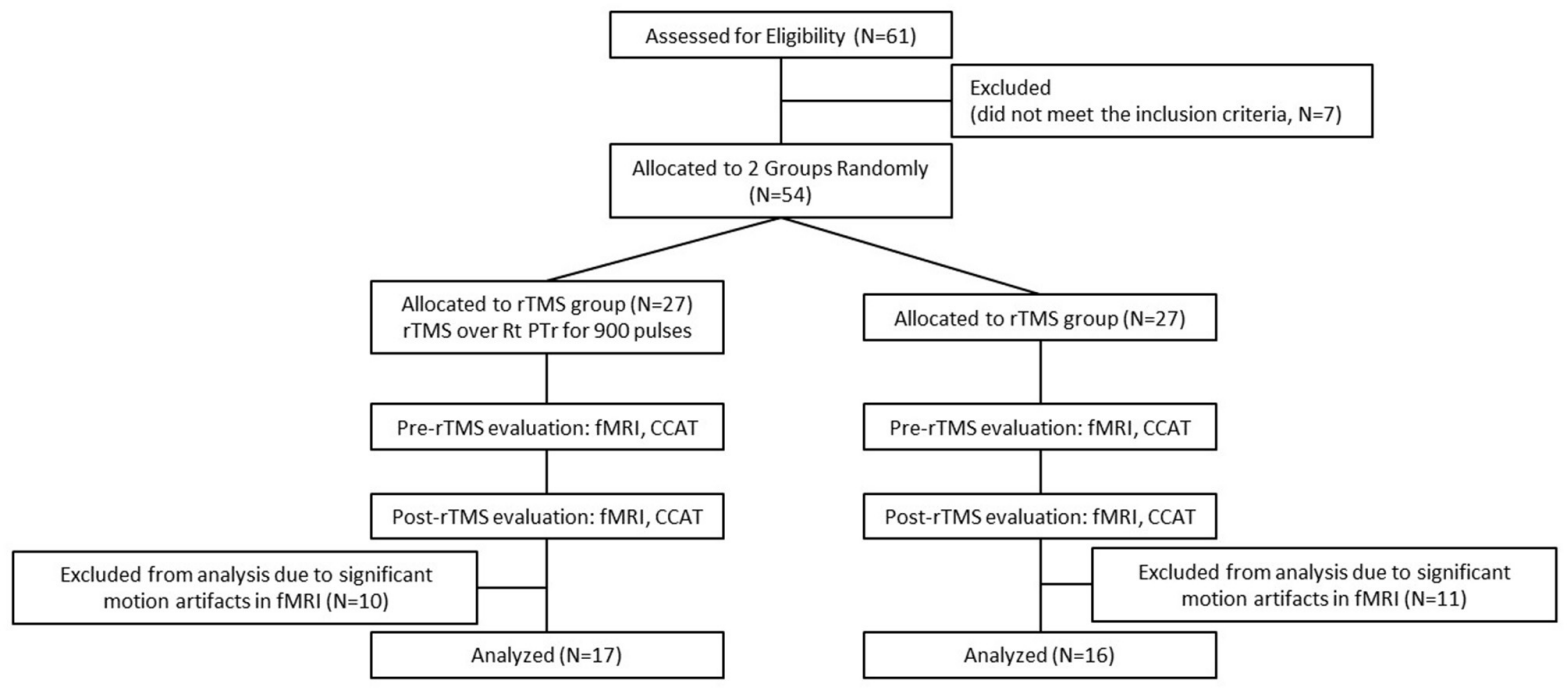
Flowchart of patient inclusion.

The enrolled patients were randomly allocated into one of the study groups (Figure 2) and either treated with inhibitory 1-Hz LF-rTMS on the contralesional pars triangularis (rTMS group, *n* = 17) for 10 daily sessions or untreated (sham group, *n* = 16). Table 1 presents the demographics and clinical assessment results of enrolled patients. No significant differences in age (*p* = 0.086, two sample *t*-test), sex (*p* = 0.805, chi-square test), and pretreatment language performance measured by the CCAT (*p* = 0.203, two sample *t*-test) were observed between the rTMS and sham groups. All the statistical analyses were performed by using the statistics toolbox in MATLAB R2020 environment.

**Table 1:** Demographic data and clinical assessment of all the subjects.

	**rTMS group (*n* = 17)**	**Sham group (*n* = 16)**	***P*-values**
Age	54.71 ± 12.03	62.94 ± 14.59	0.086
Gender (M:F)	11:6	11:5	0.805
Scores of concise chinese aphasia test	7.65 ± 2.39	6.61 ± 2.18	0.203
Months post-stroke	9.41 ± 7.27	12.63 ± 12.90	0.381

### Protocol for rTMS

We performed rTMS by using Magstim Rapid^2^ (Magstim Company, Withland, Dyfed, UK) with a 70-mm figure-of-eight coil. A Dantec keypoint electromyograph (Dantec, Skovlunde, Denmark, UK) was connected to the stimulator to record the motor-evoked potentials (MEPs) of the left first dorsal interosseous muscle. We recorded MEPs by using surface Ag/AgCl electrodes, probing the resting motor threshold (rMT) on a 9 cm × 9 cm grid over the motor cortex in the frontal area. The amplified (100 μV−1 mV/div) and bandpass-filtered (20–2,000 Hz) signals were digitized at a sampling rate of 20 kHz. Subsequently, rMT was defined as the minimum intensity at which MEPs with amplitudes of at least 50 μV could be elicited in half of the 10 consecutive trials. 3T-MRI revealed that the target area of the right posterior pars triangularis was located on the rostral part of the vertical ascending ramus of the Sylvian fissure and on the caudal part of the triangular gyrus (6, 24). Studies have indicated that the optimal stimulation location of LF-rTMS is the right posterior pars triangularis with the highest naming performance after suppression (4, 25). A frameless stereotaxic system (Brainsight, Rogue Research, Montreal, Canada) was used to coregister the stimulation target in relation to the anatomical marks of a patient's head on a real-time feedback presentation system. The employed neuronavigational system was precise to the millimeter level to locate the rTMS coil at the posterior pars triangularis (26). We applied 1-Hz LF-rTMS trains at 90% of rMT to the contralesional pars triangularis for 15 min (900 pulses) *per session*. The applied stimulation protocol was mainly based on studies by Naeser et al. (4, 5).

The stimulation protocol for the sham group was identical to that of the rTMS group, except that a placebo coil (Magstim) was used for the sham stimulation; in this approach, < 5% of the magnetic output was delivered and minimal scalp current sensation was induced. The placebo coil was identical in shape and size to the real stimulation coil and produced the same audible click on discharge as the real coil. Because none of the patients had ever undergone rTMS, they could not identify whether the stimulation was real or sham. During the initial assessment for rMT, we also used the sham coil to provide a consistent sensation as during the treatment sessions. Each patient alternately received intervention in a separate room at an appointed time to avoid conversation. Intervention for both the groups consisted of 10 daily sessions (from Monday to Friday for 2 weeks) by another TMS operator different from the one who assessed the rMT. The TMS operator was blinded to patient grouping. At the end of the first and final treatment, patients were asked to state whether they could tell if the treatment they were receiving was a real or sham stimulation. All the patients reported that they could not differentiate the type of stimulation at both the time points.

No accompanying language rehabilitation training was performed during the rTMS intervention in either the rTMS or sham groups. All the participants underwent a 40-min training program by a speech-language pathologist, who was blinded to patient grouping. The training program was performed 10 min after the rTMS intervention on Monday (once a week). The training program emphasized verbal expressive skills, including repetition, phonemic training, semantic training, naming, conversation, picture description tasks, and phrase-generation tasks.

### Language Assessment

A blinded speech and language therapist determined the language performance before and after rTMS intervention by using the CCAT (27), which was at that time the only validated Chinese aphasic assessment tool. It comprised nine subtests: simple response, expository speech, matching, auditory comprehension, naming, reading comprehension, repetition, copying, and spontaneous writing. Each item was scored from 0 to 12 and all the subtest points were averaged to obtain the total CCAT score. Two more composite scores −4-expression (calculated as the sum of the scores for simple response, expository speech, naming, and imitation writing) and 3-comprehension (calculated as the sum of the scores for matching, auditory comprehension, and reading comprehension)—were also acquired.

A paired *t*-test was used to assess the time effects on the 12 items of the CCAT (including total score and two composite scores) within each group and a two-sample *t*-test was used to identify the significant difference of change scores (post- and pre- assessment) between the rTMS and sham groups. Cohen's *d*, i.e., the standardized mean difference, was further calculated to estimate the effect size (28).


(1) $$\begin{array}{l} d=\frac{M_{rTMS}-M_{Sham}}{\sqrt{\left({S_{rTMS}}^{2}+{S_{Sham}}^{2}\right)/2}}\times \left(\frac{N-3}{N-2.25}\right)\times \sqrt{\frac{N-2}{N}}\; \end{array}$$

where *M*_*rTMS*_ and *M*_*Sham*_ are the means of the rTMS and sham groups, respectively, and *S*_*rTMS*_ and *S*_*Sham*_ are the SDs of the rTMS and sham groups, respectively. Considering the sample size (*N*) of this study was relatively small, we applied the last two terms of the equation to adjust the Cohen's d (*N* = 33 in this study) (28).

### Analysis of Resting-State FC

Magnetic resonance imaging data, including three-dimensional fast spoiled gradient recalled acquisition in the steady state (3D-FSPGR) T1-weighted images [repetition time/echo time (TR/TE): 9.4/4.0 ms and voxel size: 1.0 mm × 1.0 mm × 1.0 mm] and blood oxygenation-level dependent (BOLD) rs-fMRI images (TR/TE: 2,500/30 ms, voxel size: 3.5 mm × 3.5 mm × 3.5 mm, 200 volumes, and eye closed) were acquired on a 3T MRI scanner (GE Discovery MR750) by using an 8-channel head coil. Each patient received two MRI scans before and after the treatment to evaluate the changes in brain functional networks. The average duration between pre- and post- treatment MRI was 25.67 ± 20.89 days based on the scanner and patient's schedule (the rTMS treatment took 2 weeks). The fMRI data were preprocessed based on the standard procedures (29). The average BOLD signals were calculated from the 32 selected language areas (Supplementary Table S1) with a bandpass filter (0.01–0.1 Hz) followed by the calculation of Pearson's correlation coefficients between any pair of language areas. The r-to-z Fisher transformation was applied to ensure the normal distribution. The overlapping rates between selected language areas (excluding cerebellar regions) and stroke lesions are given in Supplementary Figure S1. We categorized the changes in FC after treatment into two conditions, namely, increase of coupling strength (including increase in synchronization and increase in antisynchronization) and decrease of coupling strength (including loss of synchronization and loss of antisynchronization) (Supplementary Table S2). We would emphasize that increase of synchronization (commonly observed in short-distance or homologous FCs) or antisynchronization (commonly found in long-distance FCs) both represents a stronger coupling strength (30). The language networks were separately examined in the following connectivity sets: (1) FCs in the left (ipsilesional) hemisphere, (2) FCs in the right (contralesional) hemisphere, and (3) interhemispheric FCs (including FCs between homologous regions and between cortical regions to the contralateral cerebellum based on the previous studies (31–33).

For comparing posttreatment and pretreatment FCs for each group (intragroup test), a paired *t*-test was conducted to examine the time effect. The significance of FC changes with control of family-wise errors was examined by using the following procedures (34). First, the FCs with *p* < 0.05 according to the *t*-test results were initially filtered out and regarded as the potential differential expressed networks between conditions in each connectivity set. Second, the candidate networks were assessed by using permutation testing to control family-wise errors. We shuffled the time/group labels for each patient 5,000 times and calculated *p*-values of the candidate networks by using *t*-tests at each shuffling *t*. The combined/representative *p*-value of the candidate network was calculated by using Fisher's method (35). We denoted the combined *p*-value at shuffling *t* as *p*_*t*_ and that without any permutation (original labeling) as *p*_0_. Finally, the permutation *p*-value was calculated by dividing the number of *p*_*t*_ that was smaller than *p*_0_ by the total shuffling time (*T* = 5,000) and permutation *p* = #(*p*_*t*_ < *p*_0_)/*T*. FC changes were considered significant if permutation *p* < 0.001.

For intergroup comparison, a two-sample *t*-test (*p* < 0.05) was first applied to identify the significant difference of coupling strength changes (post-FCs and pre-FCs). We further performed a one-way multivariate ANOVA (MANOVA) (*p* < 0.05) to investigate whether the significant difference of coupling strength changes could be identified between the two groups by considering the brain as an integrated network (36). Detailed descriptions of fMRI acquisition, imaging preprocessing, and statistical analyses are provided in the Supplementary Materials.

### Stepwise Linear Regression Analysis Between Language Improvement and Altered FCs

We further performed stepwise linear regression analysis to investigate the relationship between the altered functional networks and language improvement in the rTMS group (37). Considering that the connectivity within the language network may interact mutually and jointly influence the language abilities, the multivariate regression analysis that could model the relationship between multiple independent variables (multiple FCs in this case) and the dependent variable (the CCAT score) was applied. Only the change score (post and pre) of each significantly improved CCAT item was used as the dependent variable (response) and the change scores of significantly altered FCs were input as independent variables into the linear regression analysis. The performance of generated linear regression models was evaluated by the goodness-of-fit (based on R-square, *R*^2^) and F statistic vs. a constant model (with *p* < 0.05 as significance). The influence of each dependent variable (i.e., the change score of FC) on the prediction of language improvement was considered significant if *p* < 0.05.

## Results

### Language Performance

The changes in the CCAT scores were obtained by subtracting the pretreatment assessment scores from the posttreatment assessment scores. Results of the *t*-test revealed that the improvements in total CCAT (*P* < 0.001, *d* = 1.564), auditory comprehension (*P* = 0.027, *d* = 0.767), naming (*P* = 0.003, *d* = 1.049), imitation writing (*P* = 0.038, *d* = 0.721), 4-expression (*P* = 0.008, *d* = 0.938), and 3-comprehension (*P* = 0.045, *d* = 0.730) were significantly greater in the rTMS group than that in the sham group (Table 2). In Sections Alterations of Resting-state Functional Network After LF-rTMS and Regression Analysis Between Altered FCs and Language Improvement, we focus on unraveling the changes of FCs and their relationships with the language improvement after LF-rTMS treatment.

**Table 2:** Language performance of the studied cohorts.

	**rTMS group (*****n*** **=** **17)**			**Sham group (*****n*** **=** **16)**			**Change scores (post-pre)**			
**Items**	**pre**	**post**	***P*-values**	**pre**	**post**	***P*-values**	**rTMS group**	**Sham group**	***P*-values**	**Cohen‘s d**
CCAT total score	7.65 ± 2.39	8.42 ± 2.35	<0.001^*^	6.61 ± 2.18	6.70 ± 2.24	0.245	0.59 ± 0.31	0.09 ± 0.29	<0.001^*^	1.564
Simple response	7.00 ± 3.37	7.48 ± 3.57	0.003^*^	5.58 ± 3.02	5.72 ± 3.11	0.572	0.48 ± 0.57	0.14 ± 0.95	0.212	0.419
Expository speech	5.48 ± 2.95	6.17 ± 3.26	0.002^*^	4.24 ± 2.55	4.42 ± 2.54	0.411	0.69 ± 0.76	0.18 ± 0.86	0.079	0.598
Matching	11.1 ± 1.89	11.4 ± 1.38	0.147	11.5 ± 0.94	11.6 ± 0.78	0.295	0.26 ± 0.72	0.13 ± 0.46	0.513	0.218
Auditory comprehension	7.96 ± 2.41	8.78 ± 2.37	<0.001^*^	6.95 ± 2.71	7.14 ± 2.92	0.326	0.82 ± 0.77	0.19 ± 0.76	0.027^*^	0.767
Naming	6.86 ± 3.32	7.28 ± 3.50	0.008^*^	4.86 ± 3.14	4.76 ± 3.09	0.219	0.42 ± 0.57	−0.10 ± 0.31	0.003^*^	1.049
Reading comprehension	8.16 ± 2.87	8.85 ± 2.98	0.031^*^	7.14 ± 2.64	7.11 ± 2.91	0.924	0.69 ± 1.15	−0.02 ± 0.82	0.070	0.666
Repetition	7.26 ± 2.87	7.69 ± 2.89	0.043^*^	5.64 ± 3.36	5.73 ± 3.26	0.513	0.44 ± 0.82	0.09 ± 0.52	0.158	0.476
Imitation writing	9.54 ± 2.36	10.3 ± 2.52	0.027^*^	9.01 ± 2.79	8.98 ± 3.04	0.864	0.71 ± 1.17	−0.03 ± 0.72	0.038^*^	0.721
Spontaneous writing	6.23 ± 2.45	6.6 ± 2.51	0.007^*^	5.08 ± 2.43	5.31 ± 2.33	0.045^*^	0.43 ± 0.53	0.23 ± 0.41	0.260	0.395
4-Expression	26.6 ± 1.69	28.6 ± 12.6	<0.001^*^	20.3 ± 11.1	20.6 ± 11.0	0.502	2.03 ± 1.69	0.31 ± 1.78	0.008^*^	0.938
3-Comprehension	27.8 ± 1.87	29.4 ± 5.77	0.004^*^	25.3 ± 5.83	25.6 ± 6.27	0.424	1.58 ± 1.87	0.31 ± 1.39	0.045^*^	0.730

* *represents significant differences with p values < 0.05*.

### Alterations of Resting-State Functional Network After LF-rTMS

**Figure 3:**
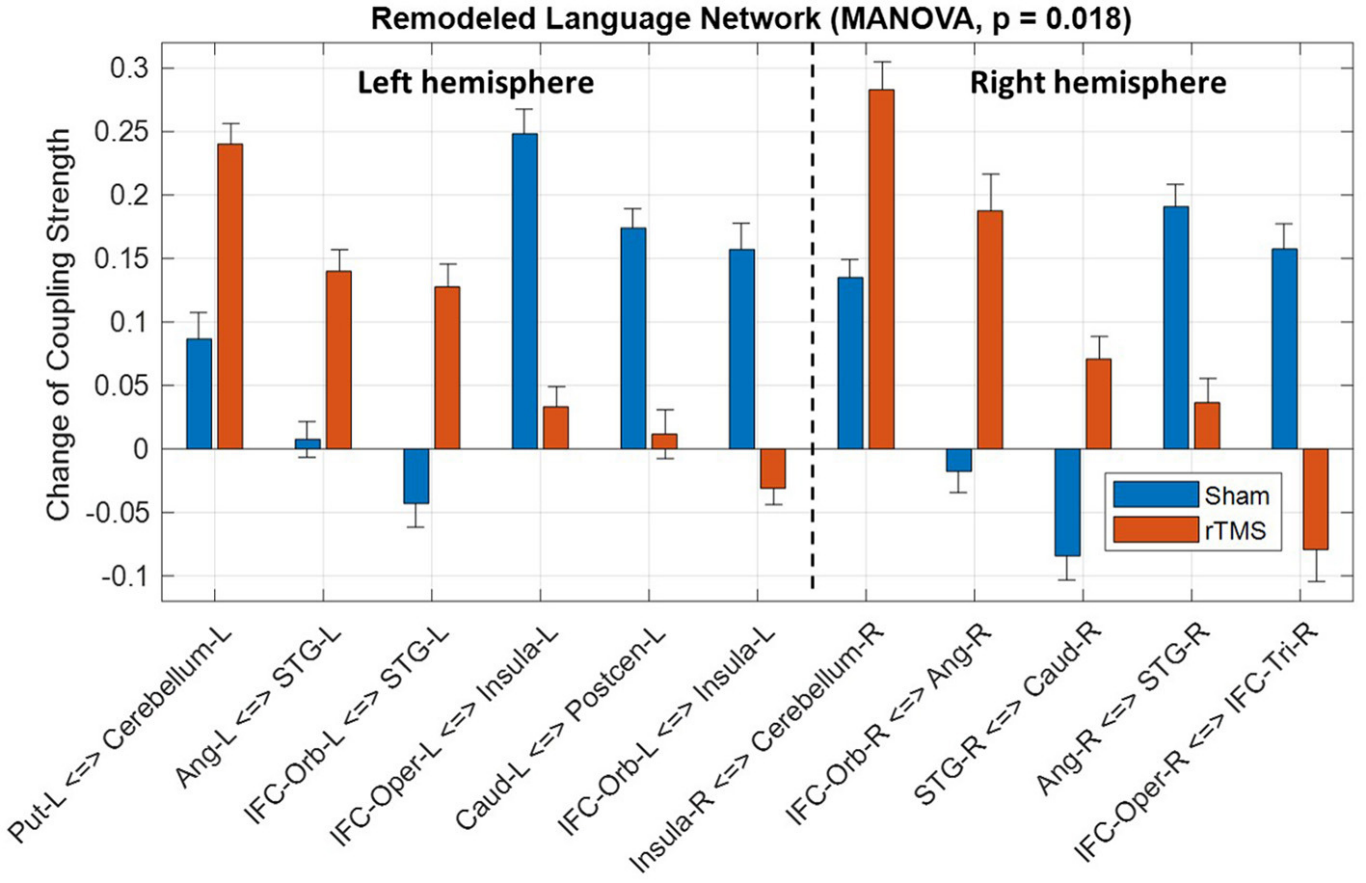
Significant difference of language network remodeling between the repetitive transcranial magnetic stimulation (rTMS) and sham groups. IFC, inferior frontal cortex; Tri, pars triangularis; Oper, pars opercularis; Orb, pars orbitalis; STG, superior temporal gyrus; Precen, precentral gyrus; Postcen, postcentral gyrus; Ang, angular gyrus; Caud, caudate; Put, putamen; L, left; and R, right.

Based on the intragroup and intergroup tests, we identified a language subnetwork with the significant difference (*p* = 0.018, MANOVA) of functional remodeling between the rTMS and sham groups (Figure 3). The identified functional remodeling involved coupling strength changes of 11 FCs (*p* < 0.05, intergroup two-sample test, Supplementary Table S4) within left and right hemispheres. In the right hemisphere, the rTMS group showed a significant decrease of coupling strength between right pars triangularis and pars opercularis, whereas the sham group presented an increase of this connectivity. We also noticed that strengthened connections between right pars orbitalis and angular gyrus and between right superior temporal gyrus and caudate were presented in the rTMS group. In the left hemisphere, several connectivities associated with the Wernicke area (superior temporal gyrus) from the pars orbitalis and angular gyrus significantly increased coupling strength after the rTMS treatment compared to the sham group.

### Regression Analysis Between Altered FCs and Language Improvement

**Figure 4:**
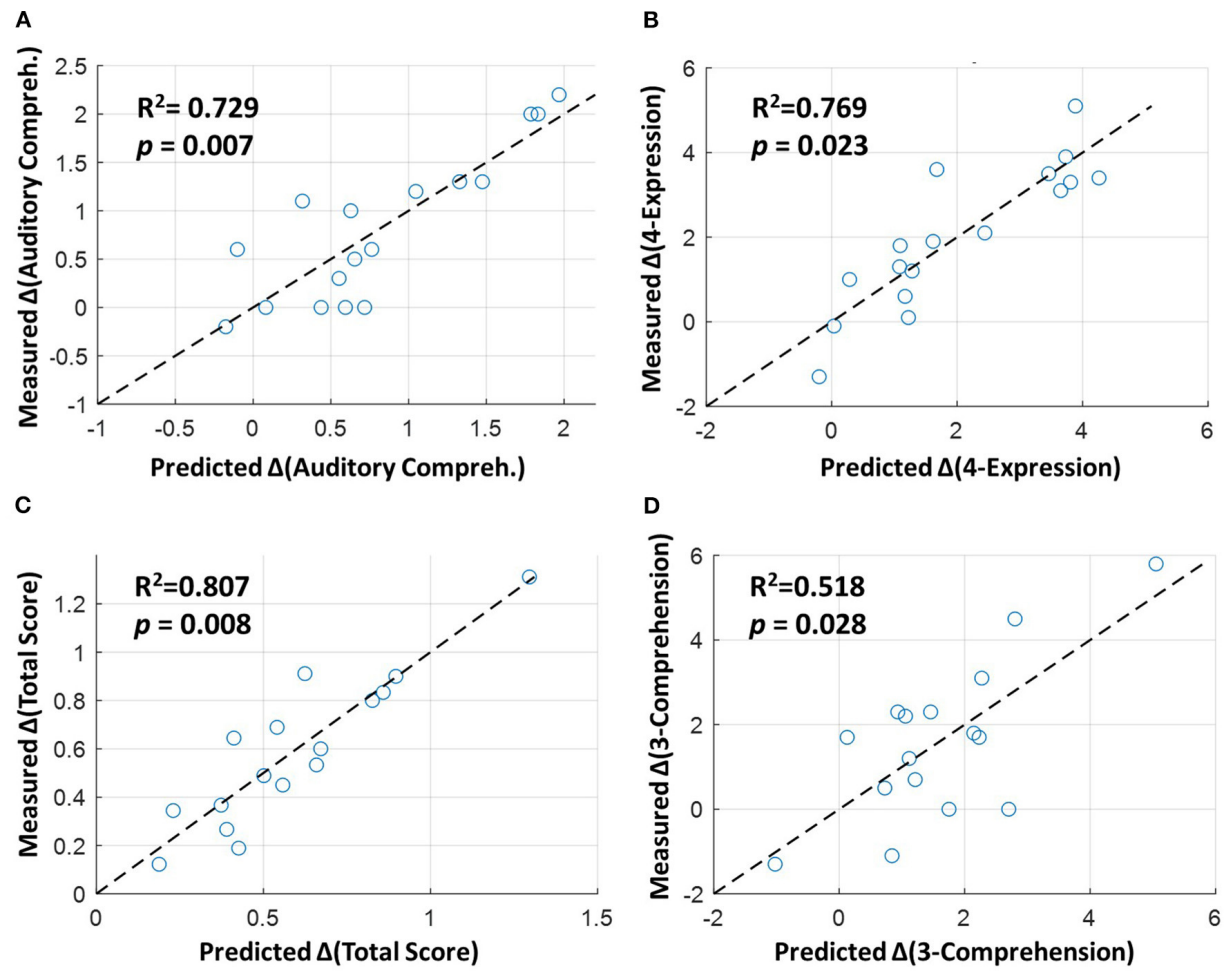
Stepwise linear regression between altered functional connectivities (FCs) and language improvement in the rTMS group. The horizontal axes represent the predicted change scores of **(A)** auditory comprehension, **(B)** 4-expression, **(C)** total score, and **(D)** 3-comprehension based on the altered FCs (given in Figure 3), and the vertical axes represent the measured/actual change scores. The dashed diagonal lines indicate the perfect prediction between the predicted and measured values based on the regression models. The detailed coefficients and *p*-values for the independent variables (FCs) are shown in Supplementary Table S5.

Among the six CCAT items with significant improvement in the rTMS group compared to the sham group (as listed in Table 2), stepwise regression analysis showed that four of them (including auditory comprehension, total score, 4-expression, and 3-comprehension) could be predicted by the change score of FCs. Specifically, the fitting performance of regression models for the auditory comprehension, 4-expression, and total score all achieved *R*^2^ > 0.729 and *p* < 0.023 (Figures 4A–C). For the 3-comprehension, the regression models showed *R*^2^ = 0.518 and *p* = 0.028 (Figure 4D). Figure 4 demonstrates that the predicted change scores of the CCAT items (ΔCCAT) based on the FC changes are consistent with the measured/actual CCAT change scores (the data points in Figure 4 locate around the dashed diagonal lines). The detailed fitting coefficients and corresponding *p-*values for each FC are given in Supplementary Table S5. All the significant correlated FCs (*p* < 0.05) with at least one CCAT item were within left or right hemispheres (i.e., intrahemispheric FCs shown in Figure 3). No interhemispheric FC showed a significant difference between the two groups or the significant correlation with the language improvement.

## Discussion

We conducted an RCT study with neuroimaging investigation to confirm the effectiveness of LF-rTMS in enhancing speech recovery and remodeling functional networks in chronic poststroke aphasia. We identified the corresponding FC modulation between right pars triangularis and pars opercularis and the circuits associated with left Wernicke area and bilateral Geschwind areas (angular gyrus), which were correlated with the language improvement after rTMS treatment. Our findings provide insights into the neuroplastic mechanisms of functional recovery in chronic poststroke aphasia. The discussion of therapeutic mechanisms of rTMS, LF-rTMS-related FC changes, and the association between functional remodeling and language recovery are addressed in the following sections.

### Mechanisms and Clinical Effects of rTMS

Both the left and right hemispheres contribute to language recovery to different extents under varied hypotheses following a stroke. Evidence from studies employing neuroimaging suggested that language recovery is primarily attributed to left hemisphere reorganization, whereas the right hemisphere assists in relevant tasks (38, 39). In the aforementioned studies, the increased activation of the right hemisphere persisted for several months following damage to the left hemisphere, which was regarded as either maladaptation or interferential compensation to the linguistic performance, whereas the activation of perilesional or spared areas in the left hemisphere facilitated language restoration during spontaneous recovery (38, 39). Stefaniak et al. proposed two potential recovery models after poststroke aphasia. The first model suggested that other cognitive networks may compensate the damaged language networks; the second model indicated that the ipsilesional and spared language-related areas may engage in the language function after the damage of primary core by the stroke (40).

Brain stimulation methods such as rTMS might alter neural plasticity and promote language restoration through long-term potentiation and long-term depression mechanisms (6, 8, 11, 15, 41). The excitatory (high-frequency) rTMS paradigm upregulates cortical excitability by facilitating synaptic transmission, whereas the LF-rTMS paradigm downregulates cortical excitability by attenuating synaptic strength (42). When applying LF-rTMS to the right homologous Broca area, specifically the pars triangularis, level B evidence revealed that LF-rTMS promoted language recovery in poststroke nonfluent aphasia through the improvement of interhemispheric imbalance from the overactive right homologous area (4, 10, 25, 39, 41, 43). Li et al. (2) reported that only naming exhibited a significant improvement after LF-rTMS treatment for mixed types of aphasia and different durations after stroke. Yao et al. (3) considered a relatively complete list of relevant LF-rTMS studies and suggested that naming, repetition, comprehension, and written language significantly improve after LF-rTMS. Their subgroup analysis further indicated that the therapeutic effects were significant among patients with chronic or acute stroke. The aforementioned meta-analyses not only confirmed the safety and treatment effects of LF-rTMS in poststroke aphasia, but also addressed the potential bias and influence of native speaker status and use of different assessment tools. In this study, we applied a well-validated assessment tool in Chinese, the CCAT, to reliably assess language functions for the recruited patients. Our results are consistent with those of studies reporting that LF-rTMS significantly improved linguistic performance in the domains of auditory comprehension, reading comprehension, naming, and verbal or writing output ability compared with baseline. Chieffo et al. (44) provided an opposite finding by comparing the effects of excitatory, inhibitory, and sham rTMS stimulation on the right inferior frontal gyrus in patients with chronic aphasia. Their results showed that only excitatory rTMS had a significant improvement of naming performance. However, this study only recruited five patients who underwent three different rTMS protocols with a 6-day washout period. The potential interactions between three different rTMS protocols were not fully addressed and the number of stimulation session was not reported.

In this study, the rTMS group exhibited favorable outcomes in terms of the total CCAT score, reflected in auditory comprehension, naming, expression, and imitation writing compared with the sham group. We demonstrated the substantial effect of rTMS in multiple domains of language skills for treating chronic poststroke aphasia.

### Low-Frequency Repetitive Transcranial Magnetic Stimulation-Related FC Changes in Chronic Poststroke Aphasia

Harvey et al. applied LF-rTMS to the contralesional par triangularis in nine patients with chronic aphasia following stroke and found a long-lasting improvement in naming coupled with activation changes in the right frontal region and left hemisphere in fMRI (15). This result indicated that LF-rTMS-mediated performance improvement may be closely linked to the alleviation of interhemispheric imbalance. However, few RCTs have employed neuroimaging to evaluate language improvement according to fMRI findings with the sham group. Furthermore, studies have mainly focused on local brain activation rather than the connectivity between brain areas. Therefore, we investigated the FC between the language-related areas and language performance to elucidate the network connectivity changes driven by LF-rTMS treatment.

In addition to the changes in local structure and functional activation after stroke, recent articles have addressed the alteration of interregional coupling measured by using FC and its association with the deficit in language functions (45, 46). Another study concluded that patients with aphasic stroke exhibit a decrease in FC between the left medial temporal and superior temporal gyri, which can be alleviated by language therapy, whereas the increase in FC between the left inferior frontal and medial temporal gyri after the therapy was associated with improvements in phonological and semantic processing of language functions (47). Furthermore, the cerebellum and basal ganglia connect with the inferior frontal and lateral temporal gyri to assist phonological processing (48).

Our results shown in Figure 3 demonstrated that the rTMS group presented distinct FC remodeling compared to the sham group. First of all, the FCs between the perilesional and spared regions in the left hemisphere, such as the connectivity associated with Wernicke area, restored their connectivity strength, whereas the right cortical areas, such as the FC between right pars triangularis and pars opercularis, reduced its coupling strength. These findings are consistent with the abovementioned literatures that the effect of LF-rTMS may suppress the dominating role of right hemisphere and, hence, restore the interhemispheric inhibition to facilitate the functional remodeling in the left hemisphere.

### Functional Remodeling Induced by LF-rTMS Supports Language Recovery

Because few studies have focused on LF-rTMS effects on patients with chronic stroke, rTMS-related neuroimaging evidence to support and elucidate language improvement remains unclear. In this study, we applied stepwise linear regression, in which we considered multiple independent variables (multiple FC changes) for investigating the relationship between functional remodeling and language improvement after rTMS treatment. Compared with the univariate correlation analysis between each FC change and language performance, the multivariate linear regression analysis is more flexible for considering the interactions between FCs and assess the comprehensive effect of network remodeling on language improvement (37). Among the six CCAT items that were significantly improved in the rTMS group compared with the sham group (Table 2), four (auditory comprehension, total score, 4-expression, and 3-comprehension) can be predicted or underpinned based on the rTMS-induced FC alterations (Figure 4). As given in Supplementary Table S5, each of these CCAT items could be predicted by multiple FCs rather than a single FC modulation with a satisfactory goodness-of-fit and significance.

The FCs selected in the regression models were associated with the Broca area, Wernicke area, Geschwind area, striatum, and cerebellum in both the hemispheres. Comprehension of spoken or written words requires the engagement of the superior temporal gyrus, whereas only the left hemisphere is involved in nonlexical phonemic recording (49). The left hemisphere plays a dominant role in tuning different types of auditory information, processing rapid acoustic transitions, and discriminating between different consonants. In addition to the core regions in cortical areas (Broca, Wernicke, and Geschwind) for language generation and comprehension, the modulating and integrating functions of the thalamus, striatum, and cerebellum are essential in language processing, especially after stroke damage (50). The thalamus and striatum are the relay centers within the corticostriatal–thalamocortical circuits that bidirectionally connect with language-related cortices (51–53). Accordingly, the altered FCs related to these circuits may be associated with language recovery after stroke.

It is worth to note that in a tonal language, such as Chinese, pitch can distinguish word meanings, while in a nontonal language, such as English, pitch is used to convey the lexical and intonation in phonetic (54). Previous studies revealed that the superior temporal gyrus participated in phonological processing to encode phonetic features (55). Our findings based on the CCAT assessment on Chinese speakers revealed that the comprehension ability was significantly improved and correlated with the FC of superior temporal gyrus after the LF-rTMS treatment. On the contrary, a previous meta-analysis that mixed studies of tonal and nontonal languages, including Chinese, English, German, and Polish, showed no significant improvement of the comprehension ability after the LF-rTMS treatment (2). Accordingly, the disparities of characteristics and neural substrates between tonal and nontonal languages should be considered to unravel the underlying mechanism of language recovery after treatments.

**Figure 5:**
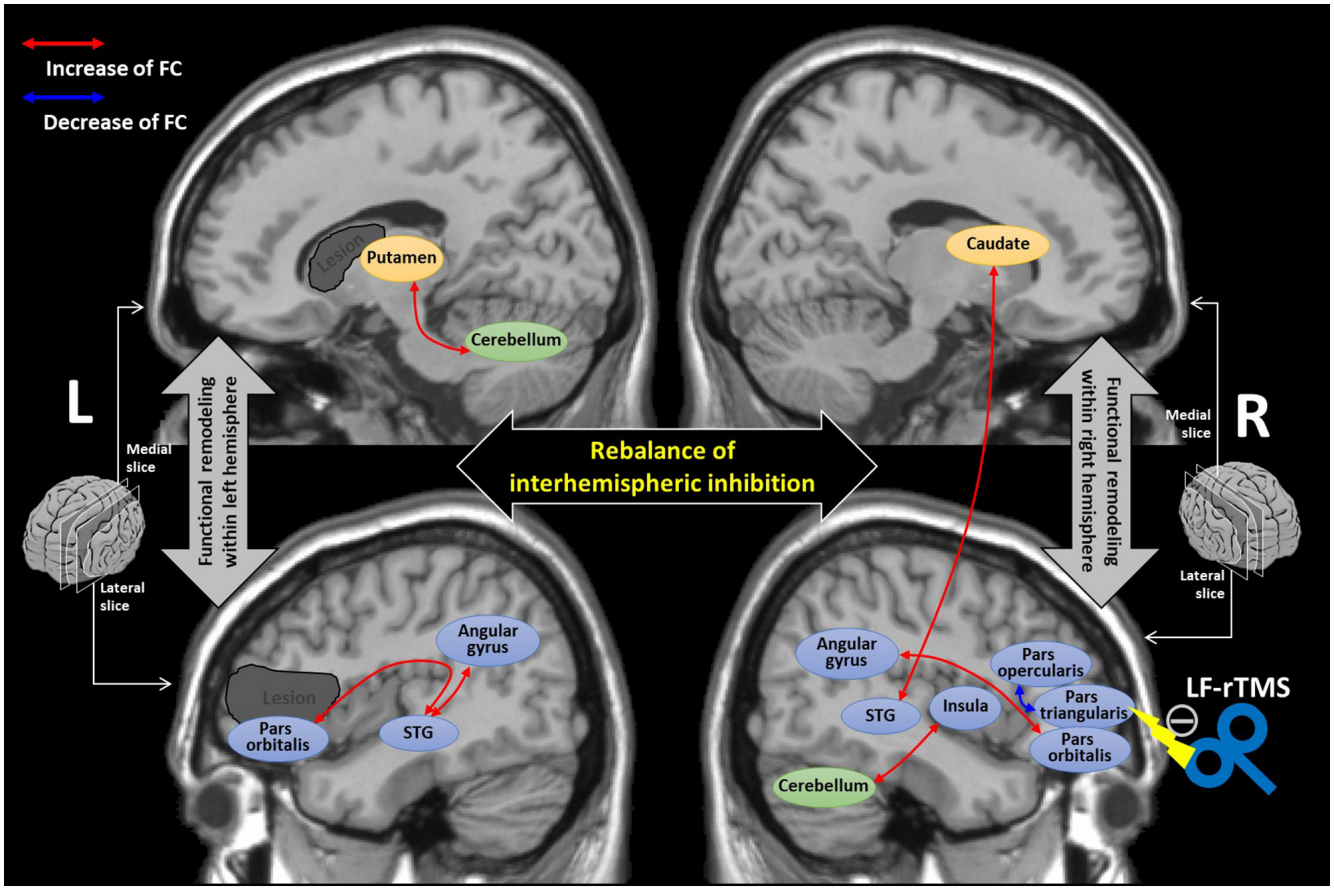
The diagram summarizing the underlying neuroplastic mechanism of language recovery after the low-frequency-rTMS (LF-rTMS) treatment. The dark gray regions indicate the potential stroke lesions mainly involved in the left inferior frontal gyrus and striatum areas. The double arrows stand for the FCs with significant correlations (identified by the regression analysis) with language improvement after the LF-rTMS treatment. The cortical areas are colored blue; the striatum areas are colored yellow; the cerebellum is colored green. STG, superior temporal gyrus.

We summarized the neuroplastic mechanism underlying the LF-rTMS treatment based on our findings in Figure 5. With the LF-rTMS inhibition on the right pars triangularis, the rebalance of interhemispheric inhibitions between two hemispheres may be restored to facilitate the network remodeling in both the hemispheres.

### Limitations and Further Considerations

Several issues of this study should be further recognized. First, this study had a relatively high exclusion rate. We excluded 21 patients because of significant head motions during the fMRI scan. Some of the patients with stroke had difficulty in fixing the head position. Despite the high exclusion rate, the final number of included participants (33 patients) was still higher than those of most prospective rTMS studies, which have enrolled fewer than 10 participants. Second, we applied a protocol with 900 pulses *per session* during the rTMS treatment instead of 1,200 pulses *per session*, as used in some other studies. This discrepancy in conditioning pulses may influence the modulation to the functional remodeling measured by the FC changes. Third, the recruited patients received language therapy once a week after the rTMS intervention. The rTMS might boost receptiveness to the language therapy session. To reduce this synergy effect, we have already reduced the frequency of language therapy to once a week. However, we were unable to completely eliminate this potential confound factor considering the patients' right to receive the language therapy. Fourth, a larger patient cohort may be required in the further study to confirm our findings. Finally, heterogeneity of lesions can be a critical factor in most stroke studies. The effects of lesion variability should be carefully addressed in the future studies.

Further considerations for the future studies might be proposed. In addition to nonfluent aphasia, LF-rTMS modulation on the right pars triangularis also resulted in concomitant improvement in language function in patients with Wernicke's aphasia in our experience. This observation suggests that fluent aphasia might benefit from this approach through the strengthening of the Wernicke-related circuits, even though the LF-rTMS target is not directly on the perceptive pathway (superior temporal gyrus).

## Conclusion

In this study, we demonstrated the promising therapeutic effects of LF-rTMS on patients with chronic aphasia following stroke. The rTMS group exhibited significant improvement in the comprehension and expression of language functions compared with the sham group. Our data further indicated that the rTMS-induced functional remodeling, involving the core language cortices and supplemental modulating areas in both the hemispheres, supported the language recovery. The engagement of the identified circuits may inspire future research on refining the rTMS protocol to improve the therapeutic effects on chronic poststroke aphasia.

## Data Availability Statement

The raw data cannot be made publicly available for ethical and legal reasons. However, researchers can submit inquiries for analyzed data to the corresponding authors upon reasonable request.

## Ethics Statement

The studies involving human participants were reviewed and approved by the local Institutional Review Board in Taipei Veterans General Hospital, Taiwan. The patients/participants provided their written informed consent to participate in this study. Written informed consent was obtained from the individual(s), and minor(s)' legal guardian/next of kin, for the publication of any potentially identifiable images or data included in this article.

## Author Contributions

C-FL and P-YT: conceptualization, funding acquisition, resources, and supervision. B-FL and C-FL: methodology and writing—original draft preparation. B-FL, C-FL, and P-YT: formal analysis and investigation. B-FL, S-CY, Y-CK, C-FL, and P-YT: writing—review and editing. All authors contributed to the article and approved the submitted version.

## Funding

This study was funded by Ministry of Science and Technology, Taiwan (103-2314-B-075-059-MY3, 107-2314-B-075-010, 106-2221-E-010-016-MY3, 108-2321-B-010-012-MY2, 108-2314-B-075-047, and 109-2314-B-010-022-MY3) and Veterans General Hospitals and University System of Taiwan Joint Research Program (VGHUST110-G7-2-2).

## Conflict of Interest

The authors declare that the research was conducted in the absence of any commercial or financial relationships that could be construed as a potential conflict of interest.

## Publisher's Note

All claims expressed in this article are solely those of the authors and do not necessarily represent those of their affiliated organizations, or those of the publisher, the editors and the reviewers. Any product that may be evaluated in this article, or claim that may be made by its manufacturer, is not guaranteed or endorsed by the publisher.
