# Supplementary material for: Functional Remodeling Associated With Language Recovery After Repetitive Transcranial Magnetic Stimulation in Chronic Aphasic Stroke
Source: Front Neurol. 2022 Mar 7;13:809843. doi: 10.3389/fneur.2022.809843 (PMC8940300; doi:10.3389/fneur.2022.809843)
Supplement: Supplementary file 1 [file Table_1.DOCX]

**Resting-state fMRI (rs-fMRI) acquisition**

Task-specific fMRI has the potential for bias due to the discrepancy of language ability between healthy subjects and patients with brain lesions; however, this is alleviated when using rs-fMRI. Because some aphasic stroke patients may not be able to perform the language task according to the instruction due to brain damage, rs-fMRI is relatively objective measurement because it does not require patients’ responses for evaluating their brain activities (Crosson et al., 2010; Fox and Greicius, 2010; Branco et al., 2016). Moreover, rs-fMRI provides a higher signal-to-noise ratio than task-related fMRI, which can reduce activity confoundment (Branco et al., 2016). Accordingly, previous studies have indicated that rs-fMRI can efficiently identify the language network (Tie et al., 2014; Branco et al., 2016; Sair et al., 2016).

**fMRI data preprocessing**

The first 10 volumes of rs-fMRI were discarded to exclude the magnetization equilibration effects and to enable adaptation of the participants to the resting state. The remaining 190 volumes were corrected for acquisition time delay among slices, and the volumes were realigned for head-motion correction (a 6-parameter affine transformation). All the corrected images were then coregistered with the corresponding anatomical T1-weighted images, followed by the spatial normalization to the T1 template image with a voxel size of 2 × 2 × 2 mm^3^. Finally, a Gaussian kernel with 6-mm full width at half maximum was used to spatially smooth the fMRI data and thereby increase the signal-to-noise ratio.

**Selected language areas for FC**

The left hemisphere is the dominant site for language functions, whereas the right hemisphere plays an assistive role in learning and comprehending speech. We selected a few brain areas associated with language functions in both hemispheres for the subsequent analysis of FC (Petrides, 2013). **Table S1** lists the Montreal Neurological Institute (MNI) coordinates of 32 selected language areas in both hemispheres. A 6-mm sphere with the center located at the specified MNI coordinate was applied for each region to extract the average BOLD signal. However, for the subcortical areas, including the putamen, caudate, and thalamus, the average BOLD signals were calculated based on the automated anatomical labeling template (AAL 116) to better delineate the structure shapes (Tzourio-Mazoyer et al., 2002).

**Meaning of Functional connectivity (FC)**

FC can be measured as a strongly positive or negative correlation depending on the anatomical distance. When two collaborated cortices are far from each other, a negative correlation is observed (Lang et al., 2012). However, once a neurological disease such as stroke occurs, the FC (either a positive or negative correlation) may vanish (i.e., the correlation coefficient may be close to 0). A preliminary study that used rs-fMRI to investigate the network changes demonstrated a decrease in FC between the left frontoparietal network and areas including right middle frontal, medial frontal, and inferior frontal gyrus in aphasic patients. The stronger the FC, the more comprehension ability was preserved (Zhu et al., 2014).

**Statistical analyses of resting-state functional connectivity**

Recent studies have suggested that functional connectivity of brain is different from other types of high-dimensional data, such as genomics, and the topological structure-related correlation between brain regions should be considered to prevent the overly conservative multiple testing adjustments (Fan et al., 2012; Chen et al., 2015). Chen et al. proposed a parsimonious differential brain connectivity network detection method to identify differentially expressed networks with significantly improved statistical power and lower false-positive rates compared to existing methods (false discovery rate and network-based statistics) (Chen et al., 2015). This detection method has been successfully applied to several resting-state fMRI studies to detect disorder-related differential networks in the digital simulations (Chen et al., 2015), autism (Chen et al., 2015; Chen et al., 2017), and schizophrenia (Chen et al., 2016; Culbreth et al., 2021). In this study, we applied the detection method using permutation testing (shuffle for 5000 times with permutation of group labels) with the control of family-wise errors to confirm whether the identified FC alterations within the differentially expressed networks after treatment were significant. The FC changes were considered significant if permutation *p* < 0.001. The detailed permutation *p* values of the altered FC networks are listed in **Table S3**.

**References**

Branco, P., Seixas, D., Deprez, S., Kovacs, S., Peeters, R., Castro, S.L., et al. (2016). Resting-state functional magnetic resonance imaging for language preoperative planning. *Frontiers in human neuroscience* 10**,** 11.

Chen, S., Kang, J., Xing, Y., and Wang, G.J.H.b.m. (2015). A parsimonious statistical method to detect groupwise differentially expressed functional connectivity networks. 36(12)**,** 5196-5206.

Chen, S., Xing, Y., Kang, J., Shukla, D., Kochunov, P., and Hong, L.E.J.a.p.a. (2016). A Network Object Method to Uncover Hidden Disorder-Related Brain Connectome.

Chen, S., Xing, Y., and Kang, J.J.F.i.n. (2017). Latent and abnormal functional connectivity circuits in autism spectrum disorder. 11**,** 125.

Crosson, B., Ford, A., McGregor, K.M., Meinzer, M., Cheshkov, S., Li, X., et al. (2010). Functional imaging and related techniques: an introduction for rehabilitation researchers. *Journal of rehabilitation research and development* 47(2)**,** vii.

Culbreth, A.J., Wu, Q., Chen, S., Adhikari, B.M., Hong, L.E., Gold, J.M., et al. (2021). Temporal-thalamic and cingulo-opercular connectivity in people with schizophrenia. 29**,** 102531.

Fan, J., Han, X., and Gu, W.J.J.o.t.A.S.A. (2012). Estimating false discovery proportion under arbitrary covariance dependence. 107(499)**,** 1019-1035.

Fox, M.D., and Greicius, M. (2010). Clinical applications of resting state functional connectivity. *Frontiers in systems neuroscience* 4**,** 19.

Lang, E.W., Tomé, A.M., Keck, I.R., Górriz-Sáez, J., and Puntonet, C.G. (2012). Brain connectivity analysis: A short survey. *Computational intelligence and neuroscience*.

Petrides, M. (2013). *Neuroanatomy of language regions of the human brain.* Academic Press.

Sair, H.I., Yahyavi‐Firouz‐Abadi, N., Calhoun, V.D., Airan, R.D., Agarwal, S., Intrapiromkul, J., et al. (2016). Presurgical brain mapping of the language network in patients with brain tumors using resting‐state f MRI: Comparison with task f MRI. *Human brain mapping* 37(3)**,** 913-923.

Tie, Y., Rigolo, L., Norton, I.H., Huang, R.Y., Wu, W., Orringer, D., et al. (2014). Defining language networks from resting‐state fMRI for surgical planning—a feasibility study. *Human brain mapping* 35(3)**,** 1018-1030.

Tzourio-Mazoyer, N., Landeau, B., Papathanassiou, D., Crivello, F., Etard, O., Delcroix, N., et al. (2002). Automated anatomical labeling of activations in SPM using a macroscopic anatomical parcellation of the MNI MRI single-subject brain. *Neuroimage* 15(1)**,** 273-289.

Zhu, D., Chang, J., Freeman, S., Tan, Z., Xiao, J., Gao, Y., et al. (2014). Changes of functional connectivity in the left frontoparietal network following aphasic stroke. *Frontiers in Behavioral Neuroscience* 8**,** 167.

**Table S1.** Coordinates of selected language areas

| **Brain regions** | **MNI coordinate of center (mm)** | | |
| --- | --- | --- | --- |
| ***Inferior frontal gyrus (Broca area)*** | **X** | **Y** | **Z** |
| Left pars opercularis | -48 | 13 | 17 |
| Right pars opercularis | 49 | 12 | 17 |
| Left pars triangularis | -47 | 27 | 6 |
| Right pars triangularis | 46 | 26 | 7 |
| Left pars orbitalis | -40 | 31 | -13 |
| Right pars orbitalis | 38 | 30 | -12 |
| ***Angular gyrus (Geschwind area)*** |  |  |  |
| Left angular gyrus | -46 | -60 | 33 |
| Right angular gyrus | 46 | -59 | 31 |
| ***Superior temporal gyrus (Wernicke area)*** |  |  |  |
| Left superior temporal gyrus | -53 | -21 | 7 |
| Right superior temporal gyrus | 54 | -19 | 7 |
| ***Anterior cingulate gyrus*** |  |  |  |
| Left anterior cingulate gyrus | -5 | 39 | 20 |
| Right anterior cingulate gyrus | 6 | 33 | 16 |
| ***Insula*** |  |  |  |
| Left insula | -42 | 4 | -1 |
| Right insula | 44 | 4 | 0 |
| ***Precentral*** |  |  |  |
| Left precentral | -64 | -7 | 18 |
| Right precentral | 64 | -2 | 18 |
| ***Postcentral*** |  |  |  |
| Left postcentral | -60 | -16 | 30 |
| Right postcentral | 60 | -12 | 32 |
| ***Cerebellum*** |  |  |  |
| Left cerebellum crus 1 | -35 | -67 | -29 |
| Right cerebellum crus 1 | 38 | -67 | -30 |
| Left Cerebellum crus 2 | -28 | -73 | -38 |
| Right Cerebellum crus 2 | 33 | -69 | -40 |
| Left cerebellum 6 | -22 | -59 | -22 |
| Right cerebellum 6 | 26 | -58 | -24 |
| Left cerebellum 7b | -31 | -60 | -45 |
| Right cerebellum 7b | 34 | -63 | -48 |
| ***Putamen (AAL atlas-based)*** |  |  |  |
| Left putamen | -24 | 4 | 4 |
| Right putamen | 28 | 5 | 2 |
| ***Caudate (AAL atlas-based)*** |  |  |  |
| Left caudate | -11 | 11 | 9 |
| Right caudate | 15 | 12 | 9 |
| ***Thalamus (AAL atlas-based)*** |  |  |  |
| Left thalamus | -11 | -18 | 8 |
| Right thalamus | 13 | -18 | 8 |

**Table S2.** Effect categories of FC changes

|  | **Effect categories** | **z values of FC** | |
| --- | --- | --- | --- |
|  |  | **Pre-treatment** | **Post-treatment** |
| **Increase of coupling strength** | Increase of synchronization | positive | more positive |
|  |  | negative | positive |
|  | Increase of anti-synchronization | negative | more negative |
|  |  | positive | negative |
| **Decrease of coupling strength** | Loss of anti-synchronization | negative | less negative |
|  | Loss of synchronization | positive | less positive |

**Table S3**. The permutation *p* values of the altered FC networks after the treatment.

| **Permutation**  ***p* values** | **Altered FCs in the left hemisphere** | **Altered FCs in the right hemisphere** | **Altered interhemispheric FCs** | **Randomly selected FCs^#^** |
| --- | --- | --- | --- | --- |
| rTMS group | 0.0008* | 0.0002* | 0.0006* | 0.9168 |
| Sham group | 0.0004* | 0.0008* | 0.0009* | 0.4172 |

*The FC changes were considered significant as P < 0.001.

^#^For the validation, we also performed the permutation testing on randomly selected FCs (rather than the FC changes with *p* < 0.05 based on the initial t test) to confirm the elimination of false-positive rates.

**Table S4**. The statistical comparisons of coupling strength changes between the rTMS and sham groups.

|  | **Changes of coupling strength**  **(post-pre FC)** | |  |  |
| --- | --- | --- | --- | --- |
|  | **rTMS group** | **Sham group** | **p values**  **(two-sample t test)** | **p value (MANOVA)** |
| IFC-Orb-L ⬄ STG-L | 0.098±0.181 | -0.035±0.185 | 0.046 | 0.018 |
| Ang-L ⬄ STG-L | 0.132±0.186 | 0.007±0.146 | 0.040 |  |
| IFC-Oper-L ⬄ Insula-L | 0.020±0.132 | 0.171±0.211 | 0.019 |  |
| IFC-Orb-L ⬄ Insula-L | -0.020±0.114 | 0.128±0.213 | 0.018 |  |
| Caud-L ⬄ Postcen-L | 0.010±0.193 | 0.152±0.167 | 0.031 |  |
| Put-L ⬄ Cerebellum-L | 0.240±0.179 | 0.087±0.231 | 0.040 |  |
| IFC-Oper-R ⬄ IFC-Tri-R | -0.079±0.279 | 0.158±0.220 | 0.011 |  |
| IFC-Orb-R ⬄ Ang-R | 0.188±0.320 | -0.018±0.186 | 0.033 |  |
| Ang-R ⬄ STG-R | 0.036±0.211 | 0.191±0.194 | 0.037 |  |
| STG-R ⬄ Caud-R | 0.071±0.198 | -0.084±0.211 | 0.037 |  |
| Insula-R ⬄ Cerebellum-R | 0.283±0.244 | 0.135±0.157 | 0.048 |  |

**Table S5.** Relationship between FC change scores and language improvement after rTMS treatment

| Target response  (Change scores of CCAT items) |  |  | Selected variables  (Change scores of FCs) | | |
| --- | --- | --- | --- | --- | --- |
|  | R^2^ | *p* values | Variables | Coef. | *p* values |
|  |  |  |  |  |  |
| Auditory Comprehension | 0.729 | 0.007 | Interaction between  IFC-Orb-L ⬄ STG-L &  Put-L ⬄ Cerebellum-L | 17.199 | 0.013 |
|  |  |  | Interaction between  Put-L ⬄ Cerebellum-L & STG-R ⬄ Caud-R | 11.039 | 0.034 |
|  |  |  | Intercept | 0.057 | <0.001 |
| 4-expression | 0.769 | 0.023 | IFC-Oper-R ⬄ IFC-Tri-R | -3.171 | 0.028 |
|  |  |  | Put-L ⬄ Cerebellum-L | -5.782 | 0.015 |
|  |  |  | Interaction between  Ang-L ⬄ STG-L &  Put-L ⬄ Cerebellum-L | 30.299 | 0.012 |
|  |  |  | Interaction between  IFC-Orb-R ⬄ Ang-R &  STG-R ⬄ Caud-R | -19.513 | 0.006 |
|  |  |  | Intercept | 2.105 | 0.012 |
| Total score | 0.807 | 0.008 | Ang-L ⬄ STG-L | 2.851 | 0.002 |
|  |  |  | IFC-Oper-R ⬄ IFC-Tri-R | -0.828 | 0.003 |
|  |  |  | STG-R ⬄ Caud-R | 1.002 | 0.010 |
|  |  |  | Interaction between  Ang-L ⬄ STG-L &  Insula-R ⬄ Cerebellum-R | -5.559 | 0.020 |
|  |  |  | Intercept | 0.500 | <0.001 |
| 3-comprehension | 0.518 | 0.028 | Interaction between  Ang-L ⬄ STG-L &  Insula-R ⬄ Cerebellum-R | 35.380 | 0.022 |
|  |  |  | Intercept | 1.787 | 0.054 |

IFC: inferior frontal cortex, Tri: pars triangularis, Oper: pars opercularis, Orb: pars orbitalis, STG: superior temporal gyrus, Ang: angular gyrus, Caud: caudate, Put: putamen, L: left, and R: right.

**Table S6.** The statistical comparisons of pre-treatment coupling strength between the rTMS and sham groups.

|  | **coupling strength at baseline**  **(pretreatment FC)** | |  |  |
| --- | --- | --- | --- | --- |
|  | **rTMS group** | **Sham group** | **p values**  **(two-sample t test)** | **p value (MANOVA)** |
| IFC-Orb-L ⬄ STG-L | -0.072±0.140 | 0.010±0.254 | 0.259 | 0. 572 |
| Ang-L ⬄ STG-L | -0.013±0.134 | 0.006±0.200 | 0.751 |  |
| IFC-Oper-L ⬄ Insula-L | 0.091±0.239 | 0.075±0.124 | 0.815 |  |
| IFC-Orb-L ⬄ Insula-L | 0.020±0.147 | 0.024±0.143 | 0.936 |  |
| Caud-L ⬄ Postcen-L | -0.072±0.160 | -0.016±0.095 | 0.228 |  |
| Put-L ⬄ Cerebellum-L | -0.0002±0.169 | -0.064±0.201 | 0.329 |  |
| IFC-Oper-R ⬄ IFC-Tri-R | 0.388±0.252 | 0.304±0.296 | 0.387 |  |
| IFC-Orb-R ⬄ Ang-R | 0.225±0.241 | 0.181±0.245 | 0.609 |  |
| Ang-R ⬄ STG-R | -0.101±0.254 | -0.051±0.151 | 0.502 |  |
| STG-R ⬄ Caud-R | 0.006±0.212 | 0.142±0.241 | 0.095 |  |
| Insula-R ⬄ Cerebellum-R | -0.054±0.195 | -0.103±0.175 | 0.460 |  |


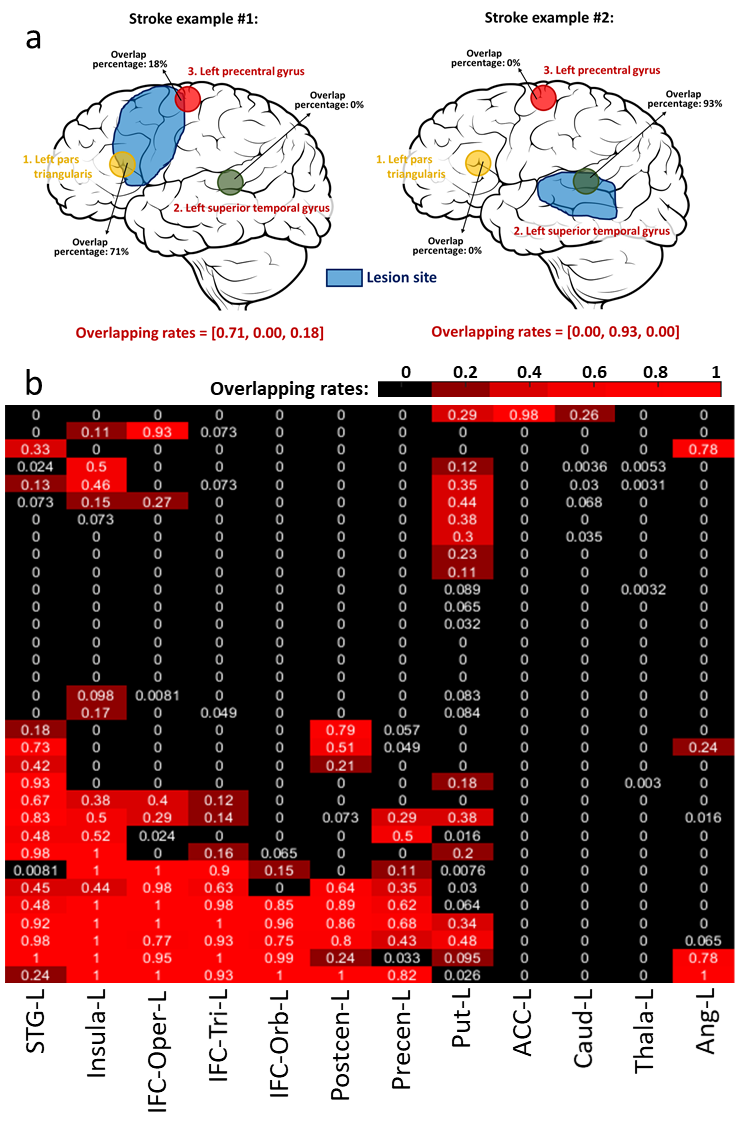


**Figure S1.** (a) Demonstration of feature vector calculation to represent the pattern of stroke lesion. Please note that only 3 (left pars triangularis, left superior temporal gyrus, and left precentral gyrus) of the 12 specified language areas are illustrated in these examples to simplify the demonstration. (b) The overlapping rates between selected language areas (excluding cerebellar regions) and stroke lesions. Each row represents the profile of overlapping rates for a patient. STG: superior temporal gyrus, IFC: inferior frontal cortex, Oper: pars opercularis, Tri: pars triangularis, Orb: pars orbitalis, Postcen: postcentral gyrus, Precen: precentral gyrus, Put: putamen, ACC: anterior cingulate cortex, Caud: caudate, Thala: thalamus, Ang: angular gyrus, L: left, and R: right.
